# Feasibility and acceptability of hazard prediction training for potential hazard prevention at Champasak Provincial Hospital, Lao PDR: a case study

**DOI:** 10.1186/s12913-025-13153-2

**Published:** 2025-08-12

**Authors:** Yumiko Maekawa, Shinsuke Murai, Khammy Souvankham, Phonepaserth Phengsackmoung

**Affiliations:** 1 Mihara General Health and Welfare Center/Mihara Health Center, Mihara Ward Office, Sakai, Japan; 2 Bureau of International Health Cooperation, National Center for Global Health and Medicine, Tokyo, Japan; 3 Nursing Department, Champasak Provincial Hospital, Pakse, Lao PDR Laos

**Keywords:** Hazard prediction, KYT, Prevention, Patient safety, 5S (Set, Sort, Shine, Standardize and Sustain)

## Abstract

**Background:**

Learning from failure is recommended for the prevention of incidence recurrence. However, a punitive climate hindered organizational learning from past failures, especially in the early stages of patient safety initiatives. Little is known about how to initiate patient safety in a punitive climate. Hazard prediction training (HPT), which focuses on healthcare professionals’ discussion of potential hazards without referencing past failures, was introduced at Champasak Provincial Hospital in Lao PDR. This study examined the feasibility and acceptability of HPTs as a potential patient safety approach under a punitive climate.

**Methods:**

In August 2019, 29 nurses were trained in HPT using four rounds of group discussions based on seven hospital scene photos to identify potential hazards, underlying factors, and priority countermeasures. A qualitative content analysis was performed on the discussion results. We analyzed differences between the 20 intended hazards and the participants’ responses, awareness of risk factors based on the P-mSHELL model, and adherence to the discussion methods.

**Results:**

The participants identified 55% (11/20) of the intended hazards. Five unintended hazards were identified mainly in complex scenes with many objects. Environmental factors were recognized most, followed by software factors, such as the absence of rules. Although patients’ families were identified as liveware factors, patient factors were overlooked. Discussions led to the identification of hazards and factors when the main subject in the photo was evident. The proposed countermeasures tended to be broad enough to cover all identified factors, and thus were abstract. Nevertheless, the result led to concrete actions such as tidying the warehouse, organizing the medicines and posting posters in the wards.

**Conclusion:**

HPTs facilitated discussions on potential hazards in a setting where discussing past failures was culturally discouraged. Findings suggest HPTs are a feasible and acceptable entry point for patient safety initiatives in similar contexts. While not addressing past failures, HPTs may raise awareness of risks and shared responsibility. Frequent recognition of environmental factors suggests synergy with 5S (sorting, setting in order, shining, standardizing, and sustaining) activities. Given the single-site design and limited sample size, further research is needed to assess the broader applicability and impact of HPTs in diverse healthcare environments.

## Background

In patient safety, learning from past failures is recommended when measures to prevent the recurrence of incidents are considered [1–4]. However, in the early stages of the development of initiatives for healthcare quality and safety, a punitive climate, in which the disclosure of failures would unintentionally result in criticisms and disciplinary actions to others, existed. A culture of blame in healthcare settings often emphasizes individual punishment, leading to fear, reluctance to report, and feelings of shame and guilt that hinder open communication and learning. Such an environment discourages healthcare professionals from speaking up about safety concerns, ultimately reducing the opportunities for organizational learning and improvement [5, 6]. Overcoming this situation requires various approaches, including the creation of a safety culture [7, 8], medical education reform [9], a focus on organizations and systems that provide healthcare [10, 11], and raising awareness among those responsible for ensuring healthcare quality and safety through collective learning opportunities [12]. However, little is known about the first step towards patient-safety initiatives in healthcare organizations where a punitive climate exists.

Lao PDR implemented a policy to improve healthcare quality in 2016 and has since intensified its efforts. However, specific policies for patient safety have yet to be established. The Japan International Cooperation Agency (JICA), in cooperation with the government of Lao PDR, implemented the Project for Improving Quality of Health Care Services in four southern provinces of Lao PDR, such as Champasak Province, from 2016 to 2021. In the project, Champasak Provincial Hospital established its Quality Improvement Committee in 2017. Its Infection Prevention and Control Committee was established the following year. Expecting a synergistic effect between this project and the efforts of Champasak Provincial Hospital, the JICA dispatched the ninth Japan Overseas Cooperation Volunteer, who was a nurse, to the nursing department of Champasak Provincial Hospital from 2019 to 2021.

The hospital’s nursing department daily observed that nurses struggled with their roles and were aware of inconveniences in their work. Incorrect dispensing and administration of medicines has occurred in the past, indicating the need for patient-safety initiatives. However, although they intended to address these issues, the hospital staff did not share failure cases. Discussing failures was perceived as a criticism of others, making it unrealistic to introduce patient safety measures through learning from errors.

In Japanese healthcare settings, efforts have been made to prevent the occurrence and recurrence, of accidents. Among these preventive measures, many hospitals practice hazard prediction training (HPT), known as Kiken Yochi training (KYT), in Japan. This healthcare safety education method enhances workers’ ability to identify and resolve risk factors, such as dangers and hazards in tasks and workplaces [13]. The HPT, initially developed in 1974, originated from the hazard prediction activities of Sumitomo Metal Industries in Japan [14]. HPT has been applied across various fields in the industrial sector as an effective safety-education program that heightens workers’ awareness of occupational hazards and reduces their unsafe behaviors [15–17]. In healthcare, HPT is distinctively aimed at ensuring the safety of not only staff but also patients [18], and as in other industries, it positively impacts the reduction of incidents or accidents [16, 19, 20]. Additionally, the HPT has been empirically shown to increase the risk sensitivity of beginners in the healthcare field, including novice nurses [21], nursing students [22–24], pharmacy students [25], and medical students [26].

The Nursing Department of Champasak Provincial Hospital decided to pilot the HPT after determining that the training method, which does not deal with past failures, would be more acceptable to the hospital staff. This study aimed to examine the feasibility and acceptability of HPT as a potential patient-safety approach in organizations where a punitive climate exists.

## Methods

### Introduction of HPT

In August 2019, the nursing department conducted half-day workshops for 29 nurses—16 from pediatrics and 13 from internal medicine—and instructed them in the four-round method of HPTs. In Round 1 of the method, the participants were asked, “What are the hazards?" (current situation assessment), to help them identify potential hazards in illustrations or photos. In Round 2, with the sentence, “this is the point of the hazards” (essence pursuit), they were asked to determine which of the identified hazards were the most significant. In Round 3, they were asked “What will we do?” (countermeasure considerations), to discuss possible solutions to critical hazards. In round 4, with the sentence, “this is what we will do” (goal setting), the team agreed on the measures that they would prioritize [27]. The nursing department encouraged participants to reaffirm their commitment to implementing measures in four rounds by finger pointing and calling.

Thereafter, the nursing department presented the participants with seven photos, each selected with specific intentions to represent typical clinical situations in Champasak Provincial Hospital where potential hazards were frequently observed:

- Photo1: Harun Bag and patient lying on the floor at the same height as the bladder, posing a risk of urine backflow or catheter malfunction.
- Photo 2: Personal belongings scattered around the bed, lack of bed rails increasing the risk of patient falls.
- Photo 3: Cleaning tools, mats for sleeping, and unused items stored together in a cluttered, unsanitary area.
- Photo 4: Patients and families living in the same room, creating crowds and placing hot items like rice cookers near flammable materials, increasing the risk of infection and burns.
- Photo 5: Disorganized medicine shelf, increasing the risk of medication errors.
- Photo 6: Mixing of used and unused syringes, increasing the risk of cross-contamination.
- Photo 7: Poor organization of ampoules, leading to potential medication errors and waste.

These photos were not intended to systematically represent general categories of hospital hazards, but to reflect real and familiar risks in the local clinical environment. The nursing department selected the photos based on their daily observations and their potential to prompt awareness and discussion of latent hazards.

Given the cultural context - where discussing past failures is often avoided due to a punitive climate – the photos were selected to encourage forward-looking dialogue focused on anticipation and prevention rather than retrospective analysis.

Although the selection may reflect contextual bias towards familiar hazards, this was a deliberate design choice to ensure ecological validity and to capture the practical awareness of staff in their everyday work setting.

**Fig. 1:**
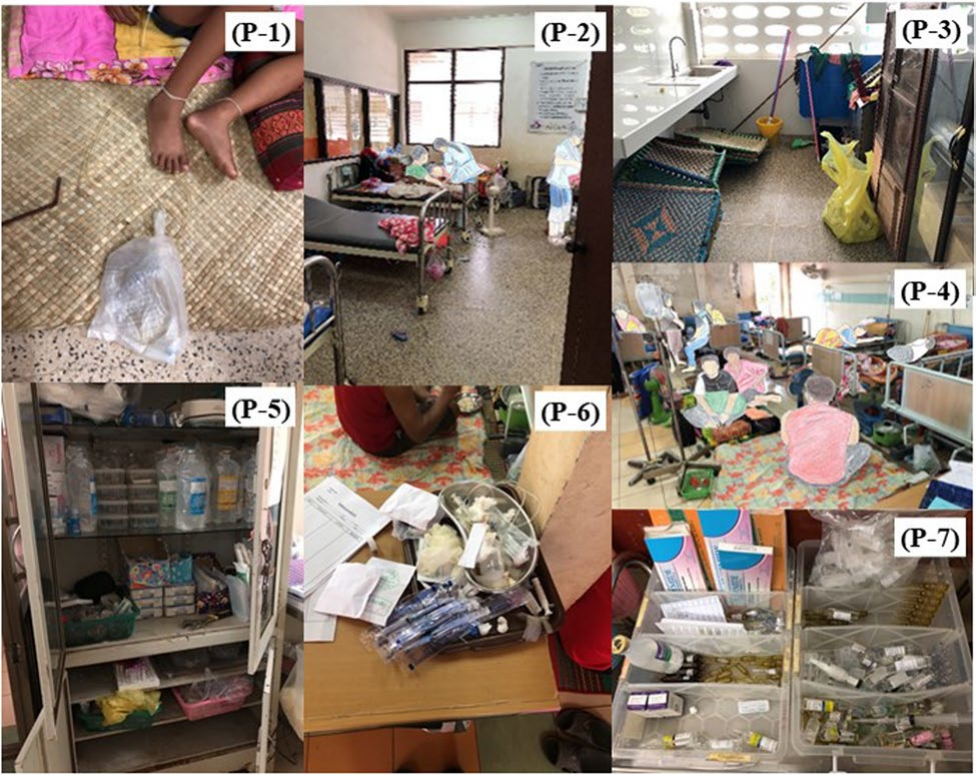
Seven hospital scene photos used in hazard prediction training (HPT). Photo 1: The patient lies on the floor in a hallway. Nearby, a Harun bag is also lying flat on the floor; Photo 2: A typical hospital ward. During hospitalization, patients’ families stay with them, eating and sleeping in the same room, with personal belongings placed around the bed; Photo 3: In a storage area, cleaning tools, temporary beds for patients, and waste are haphazardly placed together; Photo 4: Hallway outside a hospital room. Because of the shortage of beds, the floor is being used as a makeshift bed; Photo 5: A medicine shelf with pharmaceuticals and medical equipment are haphazardly arranged; Photo 6: A tray with syringes, where unused and used ones are mixed together; Photo 7: Storage of ampoules, which are not well-organized

Participants were then asked to identify potential dangers and risk factors in each photo based on the four-round method (Fig. 1).

### Data analysis

A qualitative content analysis was conducted on the discussion results of the seven HPT sessions. The analysis focused on differences between the 20 predetermined hazards identified by experienced nursing staff and those identified by participants, the types of risk factors recognized, and compliance with the four-round HPT method. 

To define and categorize hazards, we applied the P-mSHELL model [28], a healthcare-specific adaptation of Howkins’ SHEL model [29] initially conceived for the aviation industry. This framework conceptualizes hazards as arising from mismatches or vulnerabilities across seven components:

- *Patients (P)* refer to the physical, psychological and emotional conditions of patients that can affect clinical decision-making and healthcare staff performance.
- *Management (m)* includes organizational structures, leadership, and policies that shape safety culture, support risk management, and facilitate reporting systems.
- *Software (S)* refers to protocols, procedures, and standardized tools that guide healthcare practice and ensure consistency and safety.
- *Hardware (H)* includes medical equipment and tools used in clinical care, where proper design and maintenance are essential to avoid device-related errors.
- *Environment (E)* refers to physical surroundings such as lighting, noise, temperature, and layout that can influence the performance and well-being of healthcare workers.
- *Liveware for Individuals (L-I)* refers to characteristics of individual healthcare workers, such as fatigue, stress, and cognitive capacity, that influence worker performance.
- *Liveware for Others (L-O)* refers to interactions between healthcare workers and others (e.g., colleagues and patients), where teamwork and communication can influence safety.

In this study, the P-mSHELL model was not used for its original purpose of accident analysis, which focuses on identifying mismatches between the individual (Liveware for Individuals) and other components. Instead, we used it as a framework to categorize the hazards identified by participants. Following previous studies [30, 31], each identified hazard was assigned to the most relevant component to provide a comprehensive overview of the risks recognized in the local healthcare setting. The analysis did not examine the interactions or mismatches between components.

Two members of the research team improved the clarity and understanding of the discussion results, if necessary, by editing the wording to clarify the essential meaning, while endeavoring to retain the intended content of the participants’ contributions.

To ensure analytical consistency, all researchers shared and discussed the definitions and application rules of both the P-mSHELL framework and the four-round HPT method prior to analysis.

For the categorization of risk factors, two researchers independently assigned each identified hazard to P-mSHELL components based on the shared definitions. Discrepancies were discussed and resolved through consensus.

To assess adherence to the four-round method, a qualitative analysis was conducted. The aim was to examine potential deviations or adaption challenges in the Lao context, as the method was originally developed in Japan. Coding was based on agreed-upon principles, and interpretations were cross-checked through discussion.

The remaining two researchers reviewed the consolidated analysis results. All members then jointly confirmed the final categorization and coding. The final consolidated analysis results were cross-referenced with the original aggregated discussion results to ensure that all relevant input was accurately captured.

## Results

This study examined the feasibility and acceptability of HPTs as an approach for introducing patient safety initiatives for organizations with a punitive climate. The participants identified potential dangers, underlying causes, and countermeasures in the HPT on seven hospital scene photos that the nursing department considered potentially hazardous. This study analyzed the results of these discussions in terms of differences between the nursing department’s intended hazards and the participants’ responses, awareness of risk factors following the P-mSHELL model, and compliance with the four-round method. Table 1 presents the results of the HPT.

**Table 1:** Results of hazard prediction training

Photo description	Potential Dangers intended	Potential Dangers identified	Possible Underlying Causes (*P*-mSHELL components)	Recommended Actions (*P*-mSHELL components)
Photo 1: A patient is lying on the floor in a hallway. Nearby, a Harun Bag is also lying flat on the floor.	1) Retrograde infection 2) Damage to the Harun Bag	1) *Possible infection from the balloon catheter*	1) Nurses face complicated workload, which hampers their ability to perform necessary tasks efficiently (L: Individual) (L: Colleagues 1) Suboptimal healthcare environment for patients (E)	1) *Prevent hospital-acquired infections* 1) Organize the environment 1) Maintain genital hygiene 1) Improve patients’ positioning, which ensures that the Harun Bag is placed lower than the bladder by making arrangements for patients to lie in bed or on a cot.
Photo 2: A typical hospital ward. During hospitalization, the patient’s family stays with them, eating and sleeping in the same room, with personal belongings placed around the bed.	1) Presence of numerous items becomes an obstacle to care 2) Risk of falling out of the bed 3) Both the patient and family members risk tripping over objects	1) *Nursing tasks become difficult to perform* 2) Risk of the patient falling 4) Risk of patient misidentification 5) Risk of the patient contracting infections	1) The patients’ personal belongings are placed in the room (E) (L: family) 1) No rules regarding the patient’s personal belongings (S) 2) The bed lacks safety rails (H) 3) The beds are not numbered (S)	1) Collaborate with the family 5) Maintain patient safety to prevent infections in the ward 5) Provide health education to patients and their families to maintain cleanliness. • Improve air quality in the room
Photo 3: In a storage area, cleaning tools, temporary beds for patients, and waste are haphazardly placed together.	1) *Unhygienic conditions and infection risk* 2) Inaccessibility of items	1) *Risk of infection caused by the unhygienic condition of the items*	1) Lack of designated storage areas (S) 1) Improperly covered waste bins (E) (S) 1) Contamination risk in an unsanitary environment (E) (e.g., The close proximity of items such as mops, rags, waste bins for dirty items, and temporary beds for sleeping creates a high risk of contamination. For instance, cleaning tools and used patient items are placed near temporary beds.)	
Photo 4: Hallway outside a hospital room. Because of a shortage of beds, the floor is being used as a makeshift bed.	1) Risk of burns from cooking appliances (e.g., rice cookers) 2) Risk of IV stand toppling 3) Risk of falling from bed 4) Removal of indwelling needles	1) *Risk from how water’s presence* 2) Danger of IV stands falling on a head of a patient or its family members 5) Risk of infection	1) Presence of numerous family members and unnecessary personal belongings (E) 1) Hot water presents in the ward (E) (L: family) 5) Difficult work environment (E) 5) Unsanitary condition of toilets (E) • Risk of power outage (E) (H)	1, 2, 5) Educate patients and their family about the potential dangers they might face 1, 2, 5) Continuously assess daily risks while performing duties 1, 2, 5) Create understandable posters for patients and their families about potential hazards
Photo 5: A medicine shelf with pharmaceuticals and medical equipment haphazardly arranged	1) Risk of medication errors 2) Delay in response	3) Damage to Kolbens 4) Damage to medicines	3, 4) Lack of segregation rules (S)	
Photo 6: A tray with syringes, where unused and used ones are mixed together.	1) Infection due to needle stick accidents 2) Infection of patients 3) Incorrect administration of medications	1) *Risk of infection from needle piercing the skin* 2) Risk of infection for patients caused by unsanitary conditions	1) Mixing of medicines and syringes (E) 2) Unsanitary conditions within the tray (E) 1, 2) Lack of separation between waste and usable items (L: Individual) (L: Colleagues) 1, 2) No established rules for medication management (S) 1, 2) Shortage of supplies (H)	1, 2) Separate waste from usable items daily 1, 2) Establish rules for segregating waste 1, 2) Inform others through posters • Implement 5S as part of regular duties • Organize the medication shelf
Photo 7: Storage of ampoules, which are not well-organized.	1) Risk of medication errors 2) Risk of needlestick accidents 3) Risk of infection 4) Risk of injuries to hands and fingers	1) Risk of administering wrong medication to patients 2) Risk of needlestick accidents 3) Risk of infection due to the opening Metronidazole	1) Medications placed haphazardly, increasing risk of wrong administration (m) 1) Lack of proper storage rules for medications (S) 2) Needle, syringes and ampoules stored together, risking needle injuries (E) (m) 3) Improper placement of Opened antibiotics, leading to infection risks (E) (S) 3) Improper storage of antibiotics (S)	*1*,* 2*,* 3) Necessary to establish rules for the storage of medications* 1, 2, 3) The rules for medication storage should include the segregation of medications by type

Italicized: prioritized items; P: patients; m: management; S: software; H: hardware; E: environment; L (individual): liveware (individual); L (others): liveware (others). The items related to potential hazards, root causes, and countermeasures had the same leading numbers as their corresponding items. The items that were not intended by the nursing department had different numbers from the intended items. Items not prefixed with a number were discussed regardless of the previous discussion

### Potential dangers

The nursing department proposed 20 potential hazards, and the participants identified 55% (11/20) among them. In scenarios other than the medicine shelf in Photo 5, at least one potential hazard identified by the participants matched the nursing department’s intentions. Five hazards that participants recognized as potential hazards but that the nursing department did not intend were observed mainly in scenarios with multiple objects, such as patient rooms (Photos 2 and 4) and the medicine shelf (Photo 5).

The nursing department anticipated two hazards in Photo 1 and three in Photo 3; however, the participants identified only one hazard in each photo. However, excluding the medicine shelf in Photo 5, where the nursing department did not mention any intended hazard, half or more of the hazards identified by the participants matched the nursing department’s intentions. There were five unintended recognitions of potential hazards—two in the ward (Photo 2), one in the corridor in front of the ward (Photo 4), and two in the medicine shelf (Photo 5) — all in photo scenes where many objects were captured.

### Distribution of the P-mSHELL components

Table 2 presents the distributions of the P-mSHELL components of the seven scenarios. Overall, the participants most frequently identified environmental (E) factors, followed by software (S) factors, particularly observing the absence of rules (Photo 7). The participants identified the family factor in two out of four answers in the component of Liveware Others (L-O). However, the Patient component (P) was not mentioned.

**Table 2:** Distribution of the P-mSHELL components

Components	*P*−1	*P*−2	*P*−3	*P*−4	*P*−5	*P*−6	*P*−7	Total
Patient (P)	0	0	0	0	0	0	0	0
Management (m)	0	0	0	0	0	0	2	2
Software (S)	0	2	2	0	1	1	3	9
Hardware (H)	0	1	0	1	0	1	0	4
Environment (E)	1	1	2	5	0	2	2	12
Liveware: Individual (L-I)	1	0	0	0	0	1	0	2
Liveware: Others (L-O)	1	1	0	1	0	1	0	4

The participants most frequently identified environmental (E) factors, followed by software (S) factors. Family had two out of four answers for the Liveware: Others (L-O) component. However, the Patient component (P) was not mentioned (see the Results section)

### Compliance with the four-round method

#### Potential dangers and underlying causes

When there was a straightforward subject regarding potential hazards and their underlying causes, the participants quickly observed them. For example, on Photo 2, the participants observed the absence of rails on the bed, which would lead to the risk of patients falling. The participants realized that the disorganized placement of medications in Photo 7 would cause the hazard of the wrong medication being administered to patients; the storage of syringes and ampoules in the same box would cause needlestick injuries; and the opened antibacterial drugs would cause the risk of infection.

#### Underlying causes to considering priority measures

The participants used a collective expression for measures that often made their measures vague or summarized. For example, regarding Photo 1, although specific measures such as organizing the environment, keeping the genital area clean, and adjusting the height of the urine bag were suggested, they were summarized in the abstract expression of “preventing hospital infections.” This became challenging, since actions could not be specified at the level of particular places or timings with expressions such as “When doing A, let us do B.” The participants tended to develop comprehensive measures that could intervene for multiple underlying causes. For example, in Photo 4, owing to the presence of hot water and the risk of the support stand falling on a family member’s head, the dangers had to be explained to patients and those around them. Poster creation is derived as a comprehensive measure. However, it was difficult to use the expression pattern, “Because of A (risk factor), B (phenomenon) occurs,” and the consequences of the presence of hot water were not mentioned. In Photos 6 and 7, where there were no rules, the participants tended to address multiple factors by creating rules. However, in Photo 6, the consequences of being unable to sort waste were not mentioned.

Additionally, there was a case in which the essential hazards and measures were not aligned. In Photo 6, needlestick injuries were identified as essential hazards when the used and unused trays were mixed together. However, the chosen measure was not specifically aimed at preventing needlestick injuries; instead, a broader measure of waste segregation was selected. In cases such as Photos 3 and 5, the participants did not specify the measures with particular expressions even though they identified the hazards.

#### Identification of factors during the planning of measures

In Photos 1, 3, and 7, possible causes were listed during the planning of the measures. Particularly regarding Photo 1, when they were discussing the measure to ensure that the patients could lie in bed, the participants cited the reason that the height of the patient’s urine bag should be lower than the bladder, which was not currently the case.

#### Embarrassment with the finger-pointing and calling actions

The finger-pointing and calling actions, which were supposed to be implemented when the participants were deciding on priority measures, were not adopted because of the embarrassment they would cause.

### Patient safety initiatives after the pilot implementation of HPT

Based on the HPT results, the wards of Champasak Provincial Hospital took the following measures: tidying the warehouse, organizing the medicines and posting posters in the wards. Subsequently, the deputy director in charge of the nursing department stressed the need for hospital-wide implementation of HPTs. In November 2019, an HPT study session was held with 92 participants, including nurses, doctors, midwives and administrative staff from across the hospital. The following month, HPT officers were appointed to each ward; they planned measures with the nursing department and took action on activities to prevent the potential hazards identified in their HPTs. The plan was then to have regular opportunities to share practical HPT experiences within the hospital and to promote safety measures throughout the hospital. However, practical experience-sharing opportunities were not held due to the novel coronavirus infection pandemic. The JOCV had to return to Japan. In January 2020, the Patient Safety Committee of Champasak Provincial Hospital was established although the other three targeted Provincial Hospitals of the Project for Improving Quality of Health Care Services had yet considered it. The committee appointed patient safety link-staff in each department and provided regular meeting opportunities for patient safety in the hospital.

## Discussion

In the early stages of patient safety, organizations with a punitive climate had difficulty considering measures to prevent the recurrence of hazardous incidents. This study demonstrated that under such circumstances, patient-safety efforts could be initiated with HPTs that do not deal with past failure. The participants observed potential hazards and their underlying causes based on the evident subjects in the photos. They also observed that practical measures could be derived if the causes could be translated into measures. To effectively introduce HPT, which is suitable for the situation of Lao PDR, ways to implement training should be considered based on the reactions of Laotian nurses, while leveraging the basic principles of HPT [16].

### Characteristics of the responses of nurses at Champasak provincial hospital

The risks identified by the participants accounted for approximately half of the intentions of the nursing department. There are two possible reasons for this. First, the participants tended to have a fixed perspective, limiting their ideas and associations from expanding beyond specific points (as shown in their observation of Photos 1 and 3). In the case of Photo 1, where the participants discussed measures, identifying the height of the Halun bag and bladder as risk factors might have induced a bias towards having a “solution-first” approach in the nursing discipline. However, the fact that the participants had an opportunity to discuss these factors was valuable, and introducing a process in which measures could be reconsidered after the identification of factors could change the “solution-first” approach. Furthermore, the complexity of nursing tasks and an undesirable healthcare environment were identified as reasons for the nurses’ inability to provide proper services, indicating that the reasons for the lack of adequate service provision were focused on. This attention could be an entry point to address the real issues that nurses experience on the ground, with the potential for future development.

Second, while the nurses participating in the study were quick to observe that risks were directly linked to visible factors, such as those related to the environment, they rarely pointed to the risks that could arise from the actions of nurses, patients, and families that were not directly visible in the photos. Similar tendencies have been reported in Japanese studies conducted among novices with limited clinical experience [25]. However, as the nurses in Lao PDR have already accumulated clinical experience, there might have been a misunderstanding—when they were shown the photos, the participants might be thinking that they were only supposed to list the visible risks. Especially when HPT is undertaken for the first time, emphasizing the types of risks with concrete examples beforehand could help elicit participants’ thoughts and facilitate a broader examination of potential hazards and their causes from a wider perspective.

Unintended elements increased in scenes with many objects (Photos 2, 4, and 5). While the selection of themes for the illustrations used in HPT and the method of expressing hazard factors are left to the discretion of the implementer, there are at least minimal instructions, such as subdividing themes, not incorporating deliberate setups that resemble a “spot the difference” game, and not depicting too broad an area [13]. The widespread use of mobile phones with cameras has made it easier to utilize the photos of everyday scenes in healthcare settings. However, even if the intent is conveyed, the broad scope of the photos may have inadvertently captured unintended objects, drawing the participants’ attention or inducing associations related to the scene, such as a hospital ward. In Photo 4, which shows a corridor in front of a patient’s room, associations were made with issues not visible in the photo, such as dirty toilets or power outages related to the location. Although this may not accurately reflect the nursing department’s intentions, the HPT allows nurses to think freely and express their ideas. This case suggests that training faced little resistance and was a relatively acceptable approach.

Additionally, the nurses who participated in the study frequently pointed to a lack of rules as potential risks in daily operations, a software aspect, based on their observations of healthcare grounds. The early stages of undertaking initiatives for healthcare quality are when the absence of rules was recognized. This period also coincided with the time when the JICA implemented a quality improvement project for health services at the target hospital and formulated instructions for creating standard operating procedures (SOPs). HPTs can enable the examination of tasks that require rules based on actual conditions on the ground. Moreover, half of the identified liveware factors were related to patients’ families. In countries such as Lao PDR, where patient care is customarily undertaken in hospitals by patients’ families, analysing the family element from an independent perspective in advance could make it easier to identify factors stemming from families.

The tendency to choose a wide range of solutions during the development of countermeasures indicates that it is better to address many factors, particularly when intervening under resource constraints. Although this approach may seem promising at first glance, it also has the potential to postpone the consideration of more fundamental measures, necessitating a review of how discussions are conducted. There is a need to further explore how more structured decision-making processes, such as the four-round method, can be implemented in the Laotian context. The lack of “finger-pointing and calling prioritized measures” could be attributed to the absence of such a practice in their regular routines. As the objective of pointing and calling is to ensure effective implementation, exploring whether alternative methods can serve as substitutes would be helpful.

### Hazard prediction training as a starting point for patient-safety initiatives

Learning from past failures and predicting future hazards using a balanced approach that combines prevention and recurrence is desirable. By analysing past incidents, learning from actual events to prevent the recurrence of similar errors is expected, and facing accountability for errors can foster a culture of safety [32]. However, in countries such as Lao PDR where patient-safety initiatives are nascent, learning from past failures may inadvertently lead to criticism and penalties among stakeholders, which hinders the introduction of patient safety measures.

This study demonstrated that HPT aimed at prevention was acceptable in a provincial hospital in Lao PDR. Starting with hazard prediction could reduce the fear of criticism and penalties, avoiding resistance to patient-safety initiatives. By proactively devising preventive measures for potential hazards, constructive discussions can be encouraged, along with the exchange of opinions among participants [33] and learning by sharing experiences with novices [34]. These initiatives facilitate the gradual development of a trustworthy and open environment within the organization, potentially facilitating the introduction of patient safety concepts. Similarities between HPT and 5S activities (sorting, setting in order, shining, standardizing, and sustaining) have also been observed [16]. The tendency of Laotian nurses to notice environmental hazards suggests their affinity for 5S activities, which are organizational efforts to make hazardous states visible and eliminate them. Employing 5S activities to improve the environmental factors identified through HPT could be effective for the measures identified by HPT. In particular, for those engaged in healthcare ground cooperation activities such as JICA’s Japan Overseas Cooperation Volunteer nurses, 5S activities are supportive interventions. Combining 5S activities with HPT can contribute to the introduction of patient-safety initiatives.

## Limitations

This study has certain limitations. First, the potential hazards that a nursing department intends to address may not always be the correct answers for the hospital scenes on the photos. Setting correct answers in the HPT requires the use of reports on past incidents [35]. Lao PDR, the subject of this study, is in the early stages of patient safety, and this study confirmed differences only in terms of the intentions of the nursing department, which share a similar context with the participating nurses and are in a guiding position. Second, the external validity of the results was not confirmed. Our findings apply to the nursing staff of two departments in one southern provincial hospital in Lao PDR. It has yet to be verified whether our findings apply to other departments in the same hospital or other hospitals in Lao PDR. Third, unlike the illustrations in which the desired subject could be placed, some photos from the healthcare venue included many objects, making the subject unclear. This methodological limitation may have distracted the participants’ attention. Further improvements to the teaching materials could be made by asking participants about the clarity of their photos. Finally, the intervention period was short, and the participants may have needed more time to become accustomed to the four rounds of methods. HPTs have been reported to enhance participants’ safety awareness through repetition [36]. However, shortly after this intervention, the spread of the novel coronavirus disease (COVID-19) forced the suspension of the intervention in Lao PDR. As a result, the intervention period was short, and participants had insufficient time to adapt to the four-round method. This may have led to an underestimation of the acceptability of the four-round method.

## Conclusions

HPTs have the potential to promote patient-safety initiatives in healthcare organizations during the early stages of introducing patient safety. This finding implies that initiating preventive measures could facilitate patient safety efforts in other localities and organizations resistant to reporting and learning from past failures. Although a focus on prevention may seem insufficient in the short term because of potential shortcomings, such as the inability to prevent recurrences, a tendency to underestimate risks, and an avoidance of accountability for failures, in the long term, it can softly introduce a culture of safety and learning. The HPT approach may provide a foundation for preventing potential hazards, thus becoming effective as an initial step. However, the findings of this study are based on a single-site intervention with a sample size of 29 participants, which may limit the generalizability of the results to other contexts. In the future, tailoring the hazard prediction training procedures to fit cultural contexts and available resources may improve patient safety in countries, regions, and organizations facing challenges similar to those in Lao PDR. Additional studies in diverse healthcare environments are needed to confirm the broader applicability of these findings.

## Data Availability

Data is provided within the manuscript.

## Abbreviations

5S: Sort, Set in order, Shine, Standardize, and Sustain
COVID-19: Coronavirus Disease 2019
HPT: Hazard Prediction Training
JICA: Japan International Cooperation Agency
JOCV: Japan Overseas Cooperation Volunteers
KYT: Kiken Yochi Training (Hazard Prediction Training)
Lao PDR: Lao People’s Democratic Republic
QI: Quality Improvement
SOPs: Standard Operating Procedures
